# Environmental, biological and anthropogenic effects on grizzly bear body size: temporal and spatial considerations

**DOI:** 10.1186/1472-6785-13-31

**Published:** 2013-09-08

**Authors:** Scott E Nielsen, Marc RL Cattet, John Boulanger, Jerome Cranston, Greg J McDermid, Aaron BA Shafer, Gordon B Stenhouse

**Affiliations:** 1 Department of Renewable Resources, University of Alberta, Edmonton, Alberta T6G 2H1, Canada; 2 Canadian Cooperative Wildlife Health Centre, University of Saskatchewan, Saskatoon, Saskatchewan S7N 5B4, Canada; 3 Integrated Ecological Research, Nelson, BC V1L 5T2, Canada; 4 Arctos Ecological Services, Hinton, Alberta T7V 1H9, Canada; 5 Department of Geography, University of Calgary, Calgary, Alberta T2N 1N4, Canada; 6 Department of Ecology and Genetics, Uppsala University, Uppsala, SE 75240, Sweden; 7 Foothills Research Institute, Hinton, Alberta T7V 1X6, Canada

**Keywords:** Bear, Silver spoon, Environmental effects, GPS radiocollar, Temporal and spatial heterogeneity

## Abstract

**Background:**

Individual body growth is controlled in large part by the spatial and temporal heterogeneity of, and competition for, resources. Grizzly bears (*Ursus arctos* L.) are an excellent species for studying the effects of resource heterogeneity and maternal effects (i.e. silver spoon) on life history traits such as body size because their habitats are highly variable in space and time. Here, we evaluated influences on body size of grizzly bears in Alberta, Canada by testing six factors that accounted for spatial and temporal heterogeneity in environments during maternal, natal and ‘capture’ (recent) environments. After accounting for intrinsic biological factors (age, sex), we examined how body size, measured in mass, length and body condition, was influenced by: (a) population density; (b) regional habitat productivity; (c) inter-annual variability in productivity (including silver spoon effects); (d) local habitat quality; (e) human footprint (disturbances); and (f) landscape change.

**Results:**

We found sex and age explained the most variance in body mass, condition and length (*R*^2^ from 0.48–0.64). Inter-annual variability in climate the year before and of birth (silver spoon effects) had detectable effects on the three-body size metrics (*R*^2^ from 0.04–0.07); both maternal (year before birth) and natal (year of birth) effects of precipitation and temperature were related with body size. Local heterogeneity in habitat quality also explained variance in body mass and condition (*R*^2^ from 0.01–0.08), while annual rate of landscape change explained additional variance in body length (*R*^2^ of 0.03). Human footprint and population density had no observed effect on body size.

**Conclusions:**

These results illustrated that body size patterns of grizzly bears, while largely affected by basic biological characteristics (age and sex), were also influenced by regional environmental gradients the year before, and of, the individual’s birth thus illustrating silver spoon effects. The magnitude of the silver spoon effects was on par with the influence of contemporary regional habitat productivity, which showed that both temporal and spatial influences explain in part body size patterns in grizzly bears. Because smaller bears were found in colder and less-productive environments, we hypothesize that warming global temperatures may positively affect body mass of interior bears.

## Background

Understanding how spatial and temporal heterogeneity of environments affect life-history traits and the growth of individuals has been a central theme in ecology and population biology [1-3]. Among other measures of phenotype, body size for many species is highly variable across different spatial and temporal scales, which illustrates the importance of environmental heterogeneity on the growth of individuals and populations. Understanding how these spatial and temporal dynamics affect phenotypes is critical to helping identify and prioritize management actions for many species of special concern, especially in today’s rapidly changing world.

There is little argument that spatial heterogeneity of environments shape populations by affecting population density, fitness, dispersal and behaviour [4-6]. Indeed, such relationships are a cornerstone of landscape ecology [7,8] and habitat selection theory [9,10], and form the basis for natural-resource-management. Inter-annual variability in environments creates pulsed-resource dynamics that affect many animal populations [11-13] by affecting primary productivity [14-16] and the frequency and intensity of landscape disturbances [17,18]. For example, climatic oscillations that impact plant productivity will in turn affect primary consumer populations [1,19,20] and thus other trophic levels dependent on primary consumers [21,22]. For consumers that are specialized on fruit (frugivores), which often exhibit supra-annual variation in productivity [23,24], climate conditions can have an important effect on population dynamics and the health of animals. For example, masting events or mast failures are often signalled by climatic conditions [25-28]. On Barro Colorado Island in Panama, warm ENSO events stimulate fruit masting in tropical trees resulting in population increases of frugivore species [14,29]. Likewise, acorn production for many species of oaks in the USA and cones for spruce in Canada are known to mast synchronously across broad spatial scales [30-32] having profound effects on consumer populations [21,33,34].

Increasingly, it appears that such inter-annual variations have long-term effects on individuals, particularly for those experiencing boom or bust conditions during early life. In fact, conditions during *in utero* or natal periods can be as, or more, important than recent conditions on animal health and fitness [35-37]. This phenomenon is referred to as the “silver-spoon” effect as it emphasizes the importance of being born into “rich” environments [38]. Since resource conditions vary among years for nearly all ecosystems, populations often exhibit cohort effects that structure population dynamics [1,39]. For instance, cone production in white spruce during natal periods and temperature during *in utero* conditions had long-lasting effects on red squirrel reproductive success in the Yukon of Canada [37]. Likewise, population growth of stoats in New Zealand beech forests is dependent on masting [39].

One species that inhabits highly variable environments with limited resources relative to their dietary needs and large body size are grizzly (brown) bears (*Ursus arctos* L.) [40]. All the calories necessary to survive and reproduce are acquired in the approximately seven months that they are active prior to about five months of fasting in a den. The importance of limiting resources and phenotypic plasticity is further emphasized by nearly a 10-fold difference in adult body mass across the species’ range [41]. Most often, grizzly bears rely on the seasonal or inter-annual pulsing of high-calorie resources, such as salmon in coastal ecosystems [42-44] or hard and soft mast in interior populations [45-47]. Not surprisingly, body size in bears varies accordingly [48,49], having ramifications to both survival [43,50,51] and reproduction [48,52,53]. Given these resource demands and the existence of environmental uncertainty, grizzly bears have evolved a reproductive mechanism to compensate for these factors – the delayed facultative implantation of the fertilized egg dependent on autumn body condition [54-56]. Understanding body size-environment relations is therefore critical to understanding population processes in grizzly bears, particularly reproductive success and population growth.

Here, we evaluated the importance of six different factors on springtime body size patterns in grizzly bears of Alberta, Canada (see Table 1). The six factors we examined were: (1) regional habitat productivity; (2) inter-annual variability in productivity (e.g. silver-spoon effects); (3) habitat quality; (4) human footprint and activity; (5) rate of landscape change; and (6) density dependence. Our objective was to examine how each of these factors affected body mass, length and condition after accounting for age, sex, offspring dependence and capture effects.

**Table 1:** Environmental variables used to measure hypothesized environmental drivers of body size patterns in grizzly bears within Alberta, Canada

***Hypothesized environmental driver and measurement variable***	**Units**	**Measurement location(s)**^**§**^	**Temporal scale(s)**^**†**^
*A. Regional habitat productivity*			
Temperature (Winter, Spring, Summer)	°C	home range	1971-2000
Precipitation (Winter, Spring, Summer)	mm	home range	1971-2000
Ecosystem	categories	telemetry	*C*_*t*-1_
*B. Inter-annual environments (deviations)*			
Temperature (Winter, Spring, Summer)	°C	home range	*B*_*t*-1, *t*0, *t*+1_ &*C*_*t*-1, *t*0_
Precipitation (Winter, Spring, Summer)	mm	home range	*B*_*t*-1, *t*0, *t*+1_ &*C*_*t*-1, *t*0_
*C. Local habitat quality*			
Shrub habitat (quadratic)	%	telemetry	*C*_*t*-1_
Canopy cover (quadratic)	%	telemetry	*C*_*t*-1_
Variation in canopy cover	%	telemetry	*C*_*t*-1_
Deciduous canopy cover (quadratic)	%	telemetry	*C*_*t*-1_
Forest age (quadratic)	years	telemetry	*C*_*t*-1_
Forest age variation	years	telemetry	*C*_*t*-1_
Regenerating forest habitat (quadratic)	%	telemetry	*C*_*t*-1_
Variation in regen. forest age	years	telemetry	*C*_*t*-1_
Soil wetness (quadratic)	index	telemetry	*C*_*t*-1_
*D. Human footprint* &*activity*			
Private lands	%	telemetry	*C*_*t*-1_
Protected area	%	telemetry	*C*_*t*-1_
Mortality risk	index	telemetry	*C*_*t*-1_
Safe harbour habitat	index	telemetry	*C*_*t*-1_
Linear feature density	km/km^2^	telemetry	*C*_*t*-1_
Distance to human feature	m	telemetry	*C*_*t*-1_
Distance to active energy well	m	telemetry	*C*_*t*-1_
*E. Landscape change*			
Annual rate of habitat change	%	telemetry	*C*_*t*-1_

§ Home ranges estimated by 50% multi-annual kernels; climate variables measured at kernel centroid; † Temporal scales relate to time of measurements; *B* relates to birth year &*C* to capture year. For inter-annual variation, 2-yrs prior to and up to 1-yr following birth or 1-yr prior to and the year of capture are considered.

## Methods

### Study area

**Figure 1:**
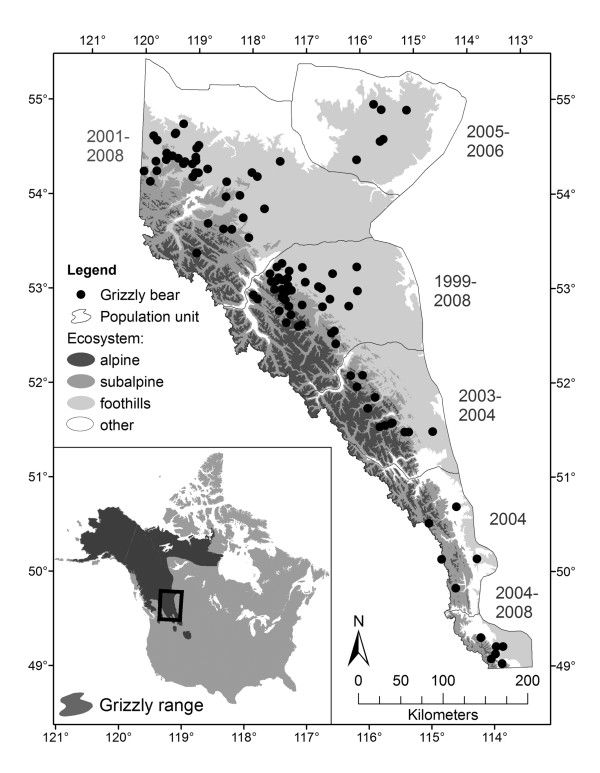
**Grizzly bear capture locations in Alberta, Canada for 112 unique animals across a 750 km distance.** Years of capture by population unit indicated along the side of each population unit. Inset map illustrates location within the current range of the species in North America.

Our study area consisted of a span of 750 km along the eastern slopes of the Canadian Rocky Mountains in Alberta, Canada (Figure 1). Grizzly bears in Alberta are considered ‘interior’ since they lack marine subsidized salmon resources. The area is characterized by cold continental climates without a dry season. Protected areas dominate the mountains, where as the foothills consist largely of multiple resource-use activities of forestry and energy extraction resulting in higher levels of forest fragmentation and human activity [57-59].

### Grizzly bear observations

**Figure 2:**
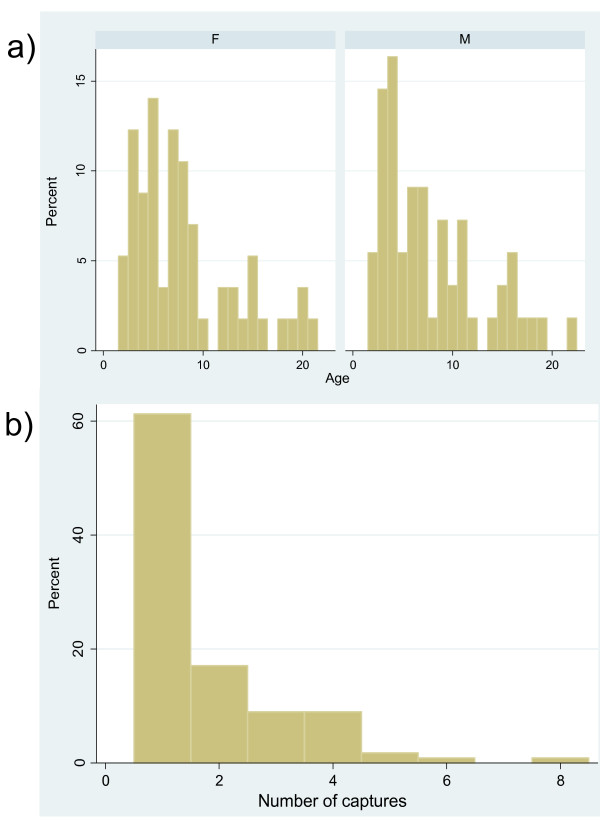
**Grizzly bear capture data for 112 animals. a)** Percent of animals, by sex, captured at each age class; **b)** Breakdown of the number of times an individual was captured (by overall percentage).

Grizzly bears were captured during the springtime (April to June) from 1999 to 2008 using remote drug delivery (Pneu-Dart Inc. or Dan-Inject) either by helicopter or following restraint by foot snares [60], and since 2004 with culvert traps [61,62]. Capture data, including a breakdown by sex, age and number of individual captures is shown in Figure 2. All bears were anesthetized using a combination of xylazine and zolazepam–tiletamine administered intramuscularly as xylazine at 2 mg/kg and Telazol at 3 mg/kg estimated body weight [63]. We administered atipamezole at 0.15–0.20 mg/kg, half-volume intramuscularly and half-volume intravenously, to reverse the effects of xylazine. Grizzly bears were weighed using a load scale (MSI-7200 Dynalink) and measured for length using a standard tape stretched over the top of the bear from the tip of the nose to the last tail vertebrae. A premolar was collected for aging bears using the number of cementum annuli [64], with adult status considered to be five years of age. For each bear a VHF ear-tag transmitter (Advanced Telemetry Systems, Isanti, MN) was attached and a Televilt Simplex, Tellus or Advanced Telemetry Systems GPS radiocollar fitted. Animal locations were transmitted every 4 hours prior to 2004 and at 1–2 hour intervals since 2004. Here we use data for 112 unique bears (57 female, 55 male) having an average age of 8.0 years (SD = 5.1) and ranging from subadult (2 years of age) to 22 years old.

We used three measures of body size to represent short- to long-term measures of growth: mass; length; and body condition. Body condition was estimated using a body condition index where mass is measured relative to length [65]. Although we had multiple capture events for some animals, we only used the most recent capture because it maximized the range of ages considered. All captures and handling were done on public lands with permits and the capture and handling procedures approved by the University of Saskatchewan’s Committee on Animal Care and Supply (Permit Number: 20010016) following guidelines provided by the American Society of Mammalogists’ Animal Care and Use Committee [66] and the Canadian Council on Animal Care [67] for the safe handling of wildlife.

### Statistical analysis

Age, sex and reproductive status (with or without offspring) of each animal was recorded. Number of times captured and density were also considered as response variables for body size measures. The local-population density was indexed as the number of genetically identified individuals surrounding a radiocollared bear [48,53]. Each bear was assigned a single geographic centroid based on their GPS telemetry locations and a buffer around this centroid based on the radius distance of the average daily movement rate of that animal’s sex-age class (4340 m to 10380 m radius). The number of detections of unique bears within each circular buffer was then estimated from DNA hair-snag information collected within 7x7km grids in 2004 to 2008. These counts were divided by the proportion of the buffer overlapping the DNA survey grid, and by the probability of capture (derived from data of the closest observed distance of GPS collared bears to known bait sites – see 67), which varied by the age, sex and reproductive status of the individual being detected, and the DNA survey stratum [68].

Regional environmental productivity was estimated for each bear at their home range centroid location based on monthly temperature and precipitation normal (or average) from 1971 – 2000 estimated with the software ClimateAB [69]. ClimateAB measures of climate normals are downscaled ANUSPLIN-interpolated monthly normal data (2.5 x 2.5 arcmin) using local weather-station data and an elevation lapse-rate adjustment [70]. Monthly climate normals for precipitation and temperature were considered for four seasonal periods (winter, spring, summer and growing season) and for the two individual months of March and July that represented late winter conditions affecting snowpack at high altitudes and peak primary productivity respectively (Table 1). We also considered ecosystem type (i.e., alpine, subalpine and foothills) as a surrogate of regional productivity based on habitat use (exposure at three possible zones of influence) measured from GPS radio-telemetry information. Zones of influence considered around each telemetry location included the local habitat-patch (HP) scale at the 30 m raster resolution, a flight-response (FR) scale of a 300 m radius representing exposure to direct human activity [71], and a landscape-encounter (LE) scale representing the average daily movement rate by sex group (scale or radius buffer).

We measured inter-annual variations in environments using ClimateAB [69] by estimating temperature and precipitation by month at each animal’s home range centroid from the time (year) prior to birth (*B*_*t*-1_) to the year of capture (C_*t*0_); due to missing data (i.e. locations prior to GPS collaring) and computational considerations, we are making the assumption that home range centroids have not changed over time or if changed that local variation in climates are small (see Discussion). The inter-annual variation (anomalies) was estimated as the absolute deviation in temperature and precipitation from 30 year (1971–2000) climate normals over the range of birth years observed in sampled bears for the same home range centroids again using ClimateAB [69]. By using anomalies rather than actual climate observations, we separated effects associated with regional productivity (climate normal) from inter-annual fluctuations (anomalies). Inter-annual variability was measured for: (1) maternal conditions (one year prior to birth; *B*_*t-1*_); (2) *in-utero* and natal conditions (birth year and yearling; *B*_*0*_ and *B*_*t+1*_); and (3) conditions during or prior to capture (*C*_t-1_ and *C*_0_) (Table 1).

Local habitat quality was measured as habitat use (GPS telemetry) at the three scales of exposure (HP, FR and LE) for nine different measures of habitat quality reflecting the association of grizzly bears with disturbed and productive environments [72-74]: canopy cover, variation in canopy cover, deciduous canopy cover, amount of shrub habitat, forest age, forest age variation, amount of regenerating forest, variation in regenerating forest age and terrain soil wetness (Table 1). Non-linear effects were considered for canopy cover, deciduous canopy cover, forest age, amount of regenerating forest used and terrain soil wetness since intermediate amounts of these habitat conditions are normally preferred [72,74,75].

We used regional measures of human footprint and activity including the amount of habitat use associated with private lands (i.e., Alberta’s whitezone; see [75]), protected areas and high- or low-risk habitats based on a mortality risk and safe harbour habitat models [58], density of linear-access features, and distance to nearest human feature or recent energy wells (Table 1). Since we did not expect body size to be affected by human features and recent energy wells beyond local effects (distances), we developed exponential decay functions for each distance variable [75] using parameters of 300 m, 1 km and 3 km. A cost-weighted distance to roads was also considered where cost was defined by terrain ruggedness (a continuous variables accounting for change in elevation) under the assumption that more rugged areas near roads would be less penetrable to humans and thus experience lower human activity. Annual rate of landscape change was measured as the annual change (%) in habitat composition using annual remote sensing of major habitat types and anthropogenic features including roads, clear-cuts and energy well-pads [76].

We used the HIREG module [77] for the software STATA 11 to estimate hierarchical regressions [78] of body size based on the six main hypothesized drivers of growth. This approach was taken in order to partition variances and test for differences among the main hypothesized factors, and account for multiple measurement variables within each hypothesized factor (block) using variable ‘blocking’ approaches. The order of hierarchical regression model considered was: (1) biology effects including density-dependence; (2) regional habitat productivity; (3) inter-annual variation in environments in the form of maternal [(year before birth), *in utero* (year of birth) and natal (year after birth)] and capture effects (year of and before capture); (4) local habitat quality; (5) human footprint; and (6) landscape change. This order reflects the need to first control for biology before examining residual variance due to environment. We chose more regional measures of environment before inclusion of local measures of environment in the hierarchical order of blocks. No interactions among blocks were considered. For each hierarchical category, we selected predictors (i.e. block of variables) based on a forward step-wise regression procedure of variable blocks using a *p* < 0.1 significance level [79]. An *F*-test was used to determine whether changes to the coefficient of variation (*R*^2^) among the main hypothesized factors for each block were significant.

## Results

### Body mass

**Figure 3:**
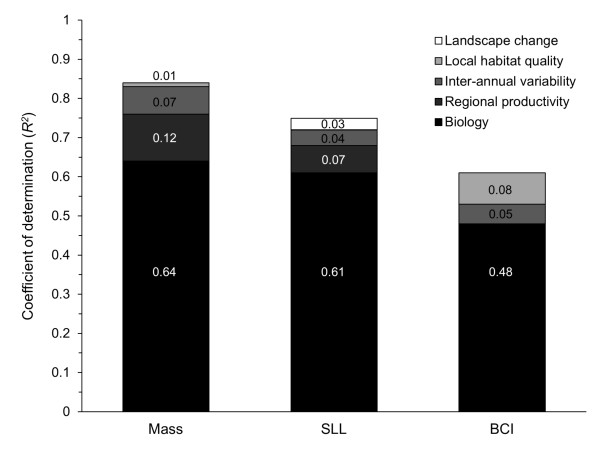
**Model coefficient of determination (*R***^***2***^**) for body mass (log[kg]), straight line length (log[SLL]), and body condition index (BCI).** Hierarchically blocked variables were partitioned to represent different hypothesized biological or environmental factors. Only significant (p < 0.05) blocked variables are illustrated.

Biological and environmental factors explained 83.5% of the variation (*R*^2^, model *F* = 50.0, *df* = 10, 68, *p* < 0.001) in body mass (Table 2, Figure 3). Age of bears was non-linearly related to mass, and the additive effect of age of male animals explained 63.5% of the total variance in springtime body mass. Regional habitat productivity explained an additional 12.4% of variance (*F* = 18.8, *df* = 2, 73, *p* <0.001), as represented by two regional measures: early spring (March) precipitation and the use (habitat patch scale) of alpine habitats (Table 2). In both cases, body mass of bears was inversely related to regional habitat-productivity measures. In addition to regional productivity, inter-annual climate variability explained an additional 6.6% of the variance (*F* = 6.5, *df* = 4, 69, *p* <0.001) and was associated with silver-spoon (maternal and natal) environments. Specifically, body mass of bears was negatively affected by anomalies in summer (July-August) temperatures in the year prior to birth. During the year of birth, anomalies in summer growing season (May-October) temperatures, winter (December-March) precipitation and August precipitation affected springtime body mass measures: specifically, body mass was higher when summer temperature and winter precipitation during the birth year were above average. Above-average August precipitation resulted in lower observed body masses (Table 2). The maternal-summer temperature was the most important effect among inter-annual climate metrics on body mass. When considering local habitats, canopy variability was inversely related to body mass, although only an additional 1% of final model variance was explained (*F* = 4.3, *df* = 1, 68, *p* = 0.043; Table 2). Body mass was not effected by the presence of cubs, number of captures, or density. Spring capture date did not have an effect (model not shown) and was therefore not included in the mass or subsequent models.

**Table 2:** **Standardized regression coefficients and significance ( *p *) of model variables describing body mass (log scale), straight line length (log scale), and body condition measures of springtime grizzly bear captures in Alberta, Canada**

***Block (hypothesized) category and measurement variables***	***Mass***		***Length***		***Body condition***	
	**StD β**	***p***	**StD β**	***p***	**StD β**	***p***
1) Biology and capture effects						
Age	1.663	<0.001	1.606	<0.001	1.898	<0.001
Age^2^	-1.348	<0.001	-1.467	<0.001	-1.450	<0.001
Adult Females (AF)					-0.367	<0.001
Adult F w/ cubs (AFC)					-0.562	<0.001
Male x Age	0.619	<0.001	0.570	<0.001		
Number of captures					-0.196	0.002
Population density						
2) Regional habitat productivity						
March precipitation	-0.255	<0.001				
Spring (May-Jun) temperature			0.202	0.002		
Alpine habitat use (HP)	-0.226	<0.001				
3) Inter-annual climate variability						
*Maternal effects (B*_*t-1*_*):*						
Summer (Jul-Aug) temperature	-0.220	<0.001	0.168	0.009		
*Natal effects (B*_*t0*_*):*						
Spring (May-Jun) temperature			0.149	0.038		
Summer (May-Oct) temperature	0.154	0.013				
Winter (Dec-Mar) precipitation	0.173	0.001				
August precipitation	-0.115	0.043				
July precipitation					-0.248	0.002
*Capture effects (C*_*t*_*):*						
4) Local habitat quality						
Canopy variation (HP)	-0.112	0.009				
Regen. forest age variation (HP)					0.288	<0.001
5) Human footprint						
6) Landscape change			0.199	0.013		

All measures of habitat use were based on global position system (GPS) telemetry data and relate to a habitat patch (HP) scale of a 30 m pixel (900 m^2^).

### Body length

Biological and environmental factors explained 75.3% of the variation (*R*^2^, model *F* = 39.0, *df* = 7, 62, *p* < 0.001) in body length (Table 2, Figfure 3). Similar to body mass, age (as non-linear quadratic function) and sex explained a large amount (61.3%) of the variation in body length. Regional-habitat productivity explained an additional 6.6% of variation in body length (*F* = 13.3, *df* = 1, 65, *p* <0.001) based on average springtime (May-June) temperatures. Bears associated with warmer spring temperatures were more likely to be longer. Inter-annual climate variability – based on maternal and natal effects – explained an additional 4.2% of variance in body length (*F* = 4.7, *df* = 2, 63, *p* <0.001). Body length was positively related to warmer summer (July-August) temperatures during maternal periods and warmer spring temperatures during the year of birth (Table 2). Habitat quality and human footprint were not related to body length, but there was a positive association with landscape change (annual rate of change in habitats associated with human disturbances) adding an additional 3.2% of model variance explained (*F* = 8.1, *df* = 1, 62, *p* <0.001). Density, number of captures and human footprint did not influence body length.

### Body condition

Biological and environmental factors explained 60.0% of model variation (*R*^2^, *F* = 14.7, *df* = 7, 68, *p* < 0.001) in springtime body condition (Table 2, Figure 3). Although body condition represents a standardized mass by length of animal, a non-linear (quadratic) age relationship with body condition was still apparent. Adult females were more likely to have a lower body condition than subadult or adult male bears, and this relationship was more pronounced if a female had cubs. Bears captured multiple times were in lower body condition than bears captured only once. Overall, the biological (including capture effects) base model accounted for 47.7% of the variance in body condition. Unlike mass and length measures, regional productivity did not affect body condition. Effects of inter-annual climate variability were observed with higher-than-normal July precipitation during the year of birth inversely related to body condition (Table 2):, this accounted for an additional 4.5% of the remaining model variance (*F* = 6.5, *df* = 1, 69, *p* = 0.013). Local habitat quality, as measured by use of habitats containing greater variation in regenerating forest age, was positively related to observed body condition (Table 2) and explained an additional 7.8% of model variation (*F* = 13.3, *df* = 1, 68, *p* = 0.001). Density of bears, human footprint, and landscape-change were not related to body condition.

## Discussion

### Biological factors and body size

Measurements of body mass and length of grizzly bears in Alberta were strongly dependent on intrinsic biological factors: age (positive, non-linear relationship) and sex (males > females). Age, sex and offspring dependence were important factors affecting body condition, which is a short-term measure of growth. Adult females, and especially adult females with cubs of the year, were likely to be in poorer condition than male bears. A negative effect of capture history (number of captures) was also observed for body condition measures which is consistent with previous observations [61]. Although population density (density dependence) is known to inversely affect body-size patterns in animals [80-82], no density dependent effects on body size patterns of grizzly bears were observed in our study. Grizzly bear populations in Alberta are likely to be below carrying capacity given locally high rates of human-caused mortality [83,84], and were recently classified by the province as ‘threatened’ given the low observed population densities [75]. This is in contrast to brown bears in Sweden that are considered healthy [85], but where body sizes of adult female bears are inversely related to population density [48].

### Temporal and spatial environmental heterogeneity

Environmental heterogeneity is an important mechanism by which animal populations are regulated [86]. Here, we found that regional heterogeneity in habitat productivity was a moderate predictor of body size patterns of grizzly bears in Alberta. The smallest bears by mass and length occurred in the least-productive and coldest environments as measured by alpine habitat use and home ranges occupying both cool average spring temperatures and high average March precipitation (snowfall). In the Canadian Rocky Mountains, all three of these factors are associated with late timing of spring snowmelt and plant emergence, which are known to affect population dynamics of other alpine mammals [87]. Since den emergence in grizzly bears in our area typically occurs in April to early May [88], the amount and timing of spring snowpack is likely a factor affecting the availability of early season food resources such as roots [89], and generally might restrict access to early spring food resources.

Inter-annual variations in climate during the years’ prior, during and/or just following birth (maternal, *in-utero* and natal environments, respectively) also affected adult body size. Such silver-spoon effects by which animals that are born into ‘rich’ conditions are favoured throughout life are consistent with observations in other mammals including polar bears [90], Soay sheep [1], red squirrels [37] and caribou [91]. Common among these studies is the importance of winter and spring climate during (natal environments) or just prior (maternal or *in utero* environments) to the year of birth, which we also observed in this study. Winter and spring climate is related to summer drought conditions in the Canadian Rocky Mountains [92], which suggests that the effect of winter and spring climate may not necessarily be directly associated with the denning period, but rather summer environments when water is limiting. We are unsure, however, how late summer precipitation affects cubs-of-the-year. It may be related to late summer food resources, such as fruit production, or affect food-resource abundance in the following year when bears are yearlings. Further, winter precipitation (December-March) anomalies during the natal birth year were positively related to body mass. We interpreted this as snow cover during winter denning providing energetic benefits (e.g. insulation) in the den for cubs of the year.

During the year prior to birth, late summer (July-August) temperature anomalies were negatively associated with body mass but positively associated with body length in grizzly bears. This late-summer environment may have affected maternal body condition prior to denning and thus subsequent condition of offspring [e.g. 53] or conversely, it may have affected the following years’ food supply during the cub-of-year period, since lag effects in fruit production are caused by weather conditions favourable to flower primordia in the mid-to-late summer period the year prior to fruiting [93]. Although we cannot be certain which factor is more important, the fact that body mass is negatively associated with late-summer temperature anomalies, where as body length is positively associated with late-summer temperature anomalies suggests to us that maternal condition is less likely (as we would expect similar responses in body mass and length if it were solely a maternal effect). Further investigations of mid and late-summer weather on pulsing in food resource abundance the following year are needed, especially in regard to the apparent opposite effects on bear mass and length.

One important consideration to our purported silver spoon effect should be discussed: that is, we have no information on our study animals prior to their first capture. This has two important implications: 1) we cannot account for litter size effects, and 2) the centroid data used to determine natal climatic conditions may not be reflective of the actual natal location. In regards to the former, not accounting for litter size should inflate the variance around our estimates. For the centroid data, this would likely only influence dispersing males, as females are philopatric [94]. For males, average dispersal distances in the province are under 50 kilometers [94], thus still largely reflective of the climate in the centroid of the current home range (differences in climates among bears are mainly regional in effect, not within populations). Further, for this limitation to bias our results, males would consistently have to disperse to poorer environments, again something we deem unlikely. Thus, we argue that the silver spoon pattern is unlikely to be altered by these factors in such away that the statistical pattern would disappear.

### Anthropogenic considerations

Human footprint did not directly relate to body size patterns of grizzly bears, but human activity indirectly affected body size by influencing habitats. The two most important measures of habitat quality were canopy closure and the age structure of forests. Bears that used habitats associated with higher canopy variability, such as forest/non-forest landscapes in the mountains or expanses of old growth forests with a recent, single-harvest sequence, had lower body masses. Conversely, bears that used forests with higher variability in regenerating forest age had higher body condition. Likewise, body length was positively related to annual landscape change. Taken together, these results suggest that human activities that fragment forests are positively associated with body size measures, although survival of bears in these environments is compromised due to high rates of human-caused mortalities [57,84]. Early successional and highly variable forests are therefore important indicators of improved habitat quality for bears given the relationship to body size patterns reported here, habitat use studies [72] and measures of food resource abundance [73,74]. We hypothesize that positive associations between body size patterns and variability in regenerating forest age are due in part to local landscape patterns in protein availability. For instance, both ungulate and ant resource use in Alberta are associated with disturbed forests [46,74].

## Conclusions

While bear body size is largely dictated by age and sex, it only accounted for about 50% of the variation. More consideration of the spatial and temporal patterns of resource availability, including the conditions early in life, is needed to better understand individual performance of animals and population dynamics. For grizzly bears in Alberta, environmental effects on body size are most affected by regional environmental gradients (space) and the environmental conditions animals are born into (time). Local-habitat heterogeneity (particularly young, patchily disturbed forests), and landscape dynamics also had a small influence on body size. It is important to emphasize that while patchily disturbed forests positively affected body size, these areas also have high rates of mortality, which could negate any positive population-level effect.

Worldwide, relationships between carnivore body size and climate warming show ambiguous trends [95]; however, polar bears body sizes have recently declined, which has been attributed primarily to loss in habitat (i.e., sea ice as a platform for hunting; [96,97]). Despite unequivocal global patterns [95], a 50 year examination of regional studies showed that carnivore body sizes have generally increased over the past half century [98]. Given the short season associated with high-alpine environments, such as the Rocky Mountains in Alberta, we hypothesize that individuals with a limited growing season and temperature-limited ecosystems, such as interior grizzly bears, might actually benefit from increases in season length associated with climate change. This prediction is largely consistent with observed body size and seasonality patterns in grizzly bears across North America [40], but may be dependent on sufficient snow cover during the denning period. In conclusion, we have demonstrated a complex interplay of biological, spatial and temporal factors on body size that collectively explained between 60 and 84% of the variation seen in Alberta’s grizzly bears.

## Competing interests

The authors declare that they have no competing interests.

## Authors’ contributions

Conceived and designed the experiments: SEN MRLC GJM GBS. Performed the experiments (animal capture and body size measurements): MRLC GBS. Contributed data/analysis tools: SEN MRLC GBS JC GJM. Analyzed the data: SEN JB MRLC JC GBS. Wrote the paper: SEN ABAS. Critically reviewed and revised paper: ABAS MRLC GBS JB JC GJM. All authors read and approved the final manuscript.
